# Mobile phone data reveals spatiotemporal recreational patterns in conservation areas during the COVID pandemic

**DOI:** 10.1038/s41598-023-47326-y

**Published:** 2023-11-20

**Authors:** Ji Yoon Kim, Takahiro Kubo, Jun Nishihiro

**Affiliations:** 1 Department of Biological Science, Kunsan National University, Gunsan, 54150 Republic of Korea; 2 Biodiversity Division, National Institute for Environmental Studies, 16-2, Onogawa, Tsukuba, 305-8506 Japan; 3 Department of Biology, University of Oxford, Oxford, OX1 3SZ UK; 4 Center for Climate Change Adaptation, National Institute for Environmental Studies, Tsukuba, 305-8506 Japan

**Keywords:** Environmental social sciences, Ecosystem services

## Abstract

Understanding visitation patterns is crucial in developing effective conservation strategies for protected areas, as it serves as an indicator for operating an ecosystem management plan that balances biodiversity and ecosystem services intertwined with public health and social benefits. However, limited data availability during the COVID-19 pandemic has hindered the comprehensive understanding of temporal changes in realized cultural ecosystem services, particularly in recreational activities within these areas. Our study utilized GPS data from mobile phones to quantify visitor characteristics and their contribution to recreational ecosystem services in protected areas at a national scale during the COVID-19 pandemic. We estimated the pandemic's relative impact on visitor patterns at 98 visitor centers in national parks and Ramsar sites in Japan. The total number of visitors and travel distance in various sizes of protected areas decreased after the outbreak of COVID-19. The number of visitors in the protected areas displayed a quick recovery despite the increasing positive COVID-19 cases during the following summer. Post-pandemic, visitors showed a preference for less densely populated protected areas closer to their home range. Our findings partly suggest that protecting a diverse range of conservation areas along the urban gradient could be an effective strategy for maintaining the resilience of recreational services during a prolonged pandemic.

Monitoring visitor numbers and activities can enhance the management capacity of protected areas. Therefore, various monitoring measures including questionnaire surveys^1^, ticket counts at the entrance, and infrared or physical counters at the tails^2^, have traditionally been implemented on site. Those traditional measures remain valuable; however, the increased risk of contagion from direct contact with visitors and the reduced human resources during the COVID-19 pandemic have limited their widespread application. In this context, visitor estimation methods using information and communication technologies (ICT) are widely tested to track visitor patterns in public spaces. One of the widely applicable measures is the use of social media, which can explore the characteristics of visitors and their interests^3^. In one example, Instagram posts were used to monitor visitor flows to World Heritage sites in Europe and North America^4^. Another example used flicker metrics to identify the structure of visitors in Germany^5^. Tenkanen and colleagues compared the different types of social media data—Instagram, Twitter, and Flickr—to gain insight into visitor activities in national parks in Finland and South Africa^6^. These recent applications can help achieve effective/efficient management; however, there have been significant setbacks in understanding visitor patterns, as data uploads are highly dependent on visitors' motivations and intentions^7^. In particular, the restrictions and changes of COVID-19 may affect their motivations, which causes unobservable bias and challenges researchers to use social media as indicators.

To address the challenges associated with self-reported ICT data, mobile phone data have recently been used in tourism and recreation studies. Human mobility derived from mobile phone GPS data has several methodological advantages for understanding visitor patterns even during the pandemic situation (Fig. 1). For example, mobile phone GPS data can capture a wide range of human mobility patterns under mobile phone coverage from local to national scales while using personal characteristics such as age and residential areas^8–11^. It also has a higher temporal and seasonal frequency of visitor counts with constant survey methods over target areas. In addition, the high flexibility of retrospective analysis using spatially explicit mobile data allows for more customized options for seasonal experimental designs^12^. In other words, the application of mobile phone data may provide more opportunities to capture the effects of unexpected changes, such as the COVID-19 pandemic.

**Figure 1:**
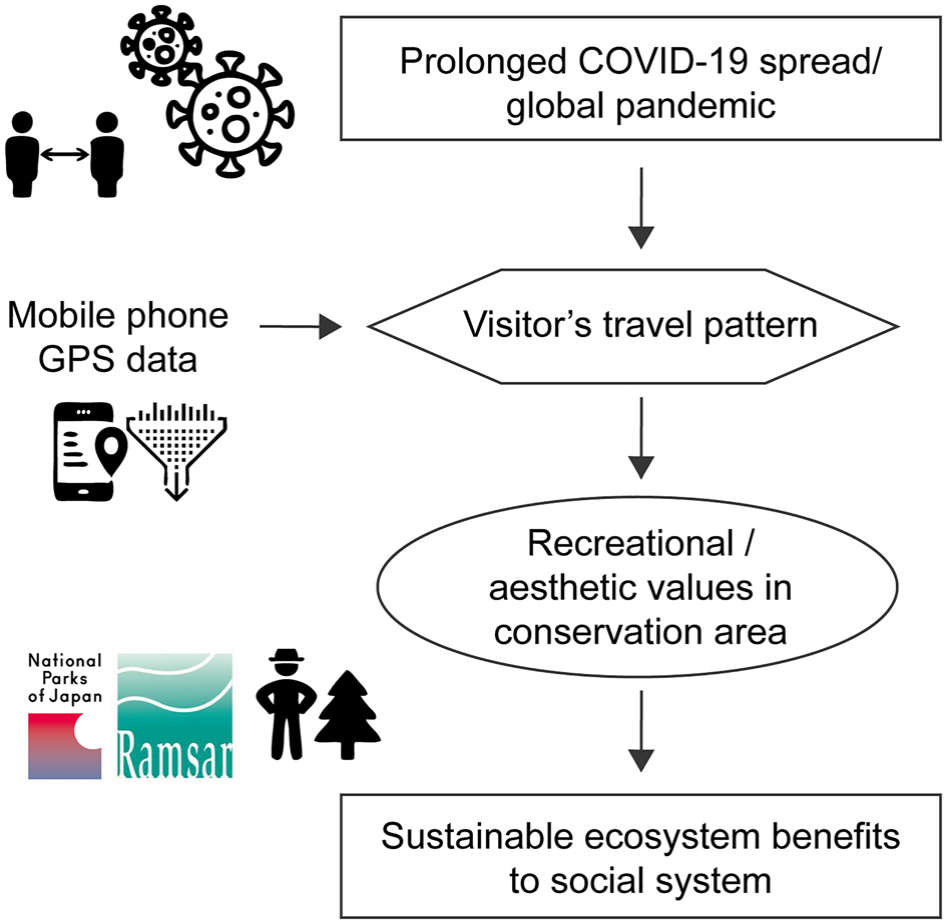
Conceptual scheme for identifying COVID-19 impacts on the use of conservation areas using mobile phone location data.

Given these advantages, recent studies have applied mobile phone big data and reported conflicting trends in human mobility and travel to the parks or regional conservation areas during the early COVID pandemic period^9,13,14^. Various types of mobile location data have been used as a tangible indicator to model the value of realized recreational ecosystem services or the potential value of different types of cultural ecosystem services^10,15–18^. However, few studies have examined the effects of seasonal changes relative to pre-pandemic conditions. To date, critical social and landscape factors associated with visitor persistence during COVID-19 have remained less understood and mostly reported as site-dependent^14,19^. In this regard, a deeper understanding of effects of COVID on protected areas use is critical for designing an adaptive management plan against increasing pandemic risks.

Conservation areas such as national parks are complex social-ecological systems comprising a variable mosaic of ecosystem types and landscapes at multiple scales^20^. The primary objectives of such areas are to protect biodiversity and ecosystem functions, and to secure a range of ecosystem services for a sustainable society. Among the diverse ecosystem services, services associated with recreational activities are the most ubiquitous and measurable cultural ecosystem services provided by natural ecosystems in conservation areas^18,21–23^. Through interactions with natural environments, people derive a variety of physical and psychological benefits from protected areas. In addition, strong interactions with natural environments tend to facilitate conservation activities and the persistence of conservation areas^24^.

However, the opportunities for such interactions do not always provided in the same form. In other words, ecosystem services are not in a static state, but in a dynamic state that changes over time depending on the climate, surrounding environment, and social conditions. For example, the daily changes of users in urban parks and the seasonal fluctuations of activities and contents in a protected area are some of the known temporal patterns of realized cultural ecosystem services (e.g., the peak of visitors in spring and fall to enjoy the flowering and leaf coloring associated with temperature changes^25^). Even with the global alliance and countermeasures to address the unprecedented COVID-19 crisis, the prolonged COVID-19 pandemic resulted in severe impacts on public health, the global economy, social activities, and, importantly, human-nature interactions^26^. From a societal perspective, the COVID-19 pandemic profoundly affected how people interact with the natural environment, which may further lead to associated changes in the perceived value associated with ecosystem services. Understanding of COVID-induced changes is an important step in developing a virtuous circle between protected areas and urban communities to improve sustainable management plans against increasing pandemic events.

Here, we have used mobile phone big data provided by a Japanese mobile phone company, KDDI, and explored the spatiotemporal pattern of visitor characteristics as a measurable indicator of recreational value in conservation areas during the COVID-19 pandemic. We integrated mobile phone data around 98 visitor centers in conservation areas (i.e., 34 national parks, 52 Ramsar sites) from 2019 to 2020. And we compared the relative change of visitor patterns along different socio-environmental conditions after the nationwide spread of COVID-19. Based on the data explicit visitor patterns, we further discussed the adaptive management strategies against social pandemic events in conservation areas.

## Results

### Visitor characteristics in social-environmental landscape

We found a general environmental setting of urban–rural landscape transition in the study sites (Supplementary Fig. 1). For example, population density and urbanized area decreased with increasing distance from the city center (Spearman’s correlation coefficient r_s_:  − 0.39; r_s_:  − 0.45). Prior to COVID-19, visitor characteristics, including number of visitors and travel distance in the protected area, both increased moderately with larger protected area size (r_s_: 0.37; r_s_: 0.29). And mean travel distance increased in the more remote protected area (r_s_: 0.67) with lower population density (r_s_: -0.52) and less urbanized area (r_s_: -0.56) in the region surrounding the visitor centers.

### Visitor dynamics during the COVID-19 pandemic

**Figure 2:**
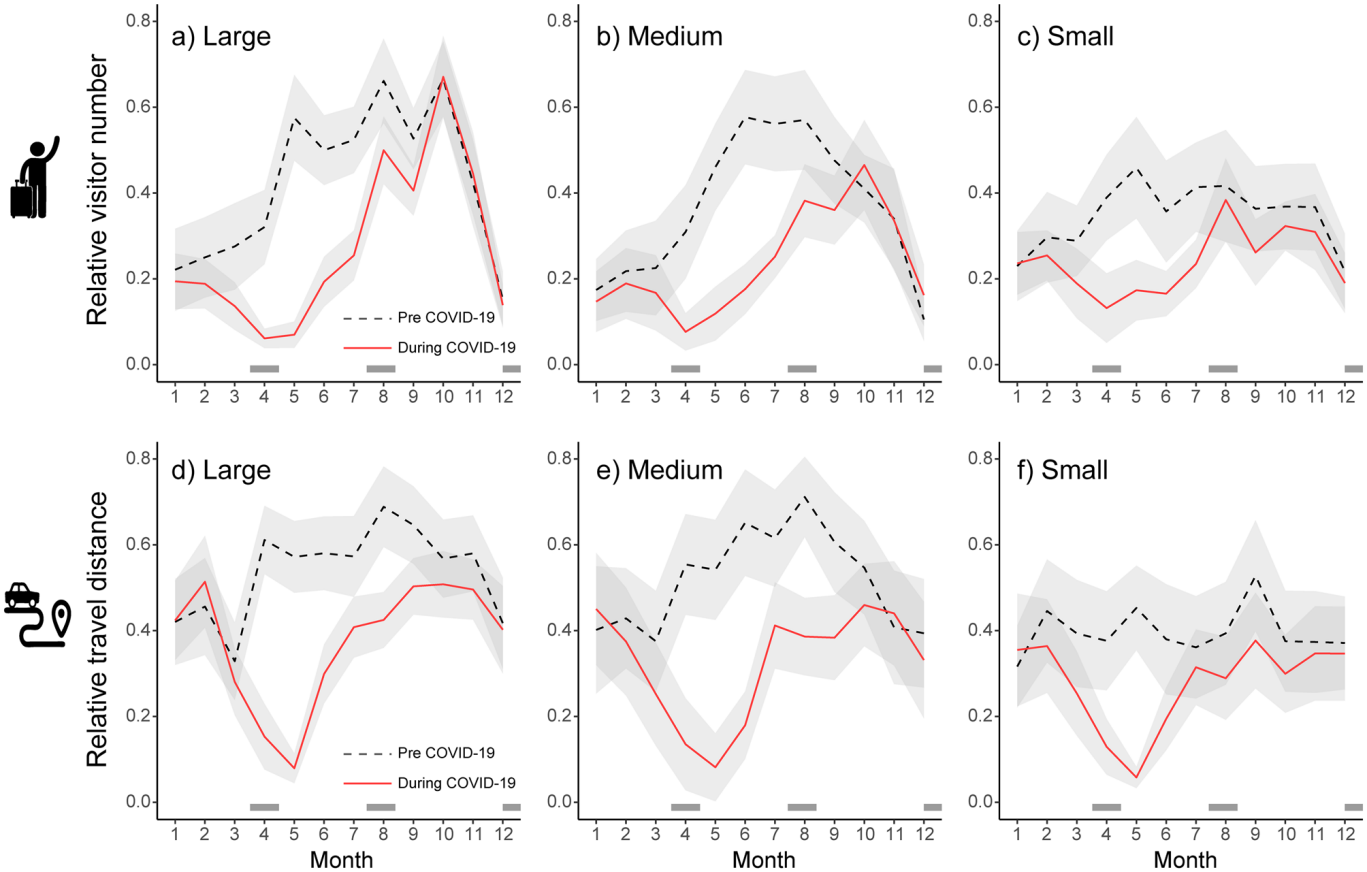
The impact of COVID-19 on the number of visitors and the travel distance to the visitor centers in the conservation area. (**a**–**c**) the relative number of visitors, (**d–f**) the relative travel distance. Size category of conservation area: a, d: large; b, e: medium; c, f: small. Pre COVID-19: Jan–Dec 2019 (dotted black line), During COVID-19: Jan–Dec 2020 (red line). Grey shading along the lines indicates the 95% confidence interval. Dark gray boxes on the x-axis indicate the peak-wave periods (i.e., 1st, 2nd and 3rd waves) of COVID-19 positive cases in the study area.

Visitor patterns recorded using mobile phone GPS data showed an abrupt decline during COVID-19 (Fig. 2a–c: number of visitors, d-f: travel distance). The relative number of visitors to the protected area after the COVID-19 pandemic changed by  − 27.7 ± 3.5% (min–max range:  − 69.7, 153.8%) compared to the pre-COVID period. The degree of visitor loss after the 1st wave of COVID-19 (Apr–May 2020) was greatest in large protected areas (mean difference:  − 50%), followed by medium ( − 40%) and small protected areas ( − 20%; Fig. 2a–c; Supplementary Table 1). Despite the increase in positive COVID-19 cases during the following summer (Supplementary Fig. 2), the relative number of visitors to the protected area showed a rapid recovery afterwards. Mean travel distance also showed a similar decreasing pattern during the first wave of COVID-19 (Fig. 2d–f). Mean travel distance changed by  − 26.0 ± 2.6% ( − 79.9, 133.2%) after COVID expansion. The recovery of relative travel distance in small reserves was slightly faster than in other size groups; after July 2020, the mean travel distance of the small reserve group recovered to similar levels as before COVID-19.

**Figure 3:**
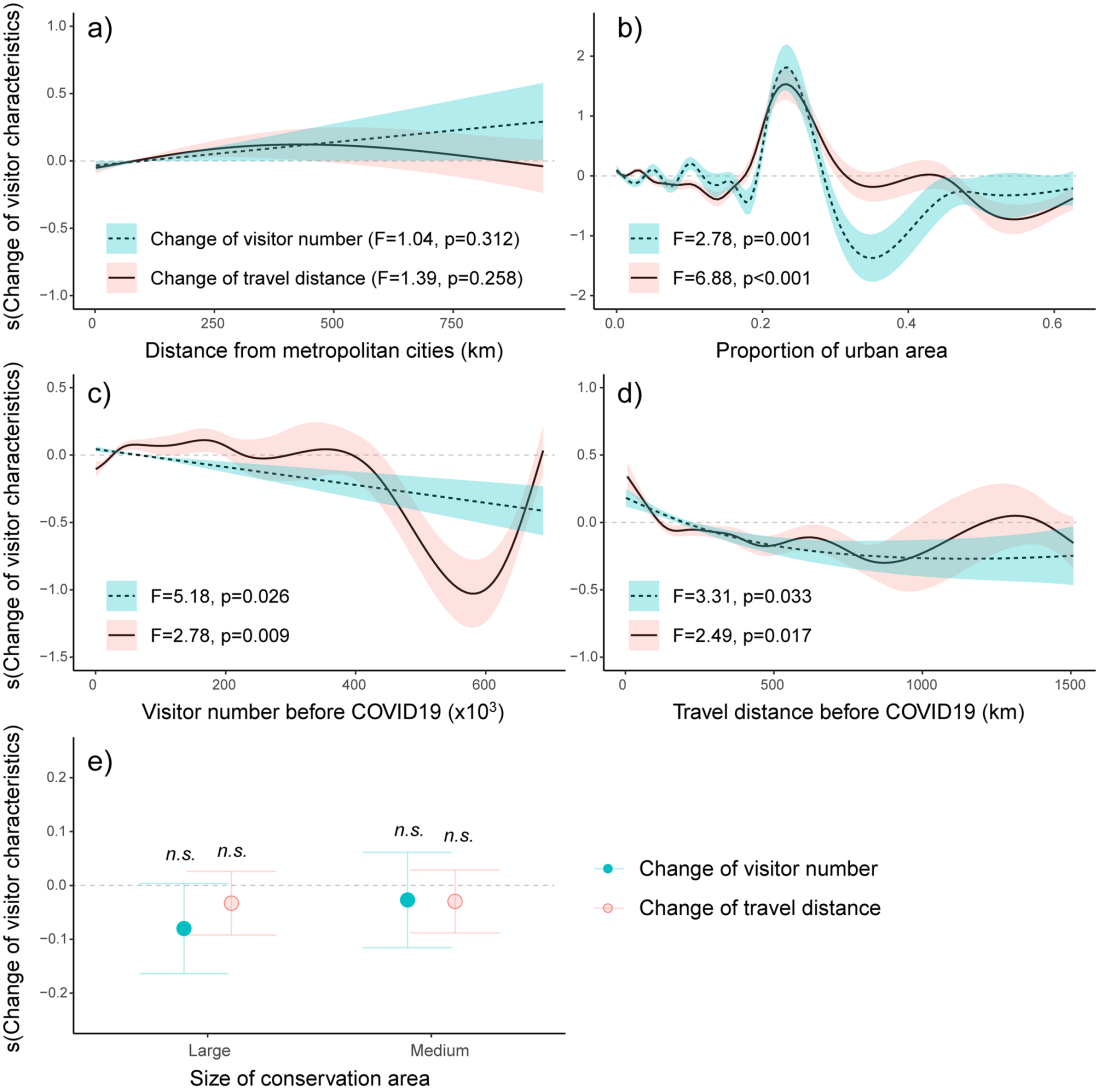
Response curves from generalized additive models (GAM) based on visitor patterns in 98 visitor centers in the national parks and Ramsar sites. Relative change in visitor numbers (dotted line) and travel distance (continuous line) to (**a**) distance from metropolitan areas, (**b**) proportion of urban area, (**c**) annual number of visitors before COVID-19, (**d**) mean travel distance before COVID-19, (**e**) size of protected area (effects compared to small size group). Colors indicate 95% confidence interval. *n.s.* not significant.

The GAMs explained the non-linear response of the relative change in visitor numbers (adjusted R^2^: 0.34) and mean travel distance (adjusted R^2^: 0.56) along a range of socio-environmental conditions during the COVID-19 (Fig. 3a–e). In particular, changes in visitor numbers during COVID-19 were more strongly related to the level of urbanization (Fig. 3b) and the level of mean visitation before COVID-19 (Fig. 3c) than to distance from urban centers (Fig. 3a). Along the urbanization gradient, the relative visitor loss was highest in the regional area with 30–40% urban coverage (Fig. 3b). The similar amount of visitor change in the urban area was shifted to the increased visitation in the less urbanized area (i.e., urban coverage: 20–30%). The number of visitors and travel distance was decreased notably in the conservation sites that had higher visitor numbers before COVID-19 (Fig. 3c). Both the number of visitors and the mean travel distance to the proximate sites (i.e., < 100 km from the visitor’s home range) were more preferred during the COVID-19 pandemic (Fig. 3d).

**Figure 4:**
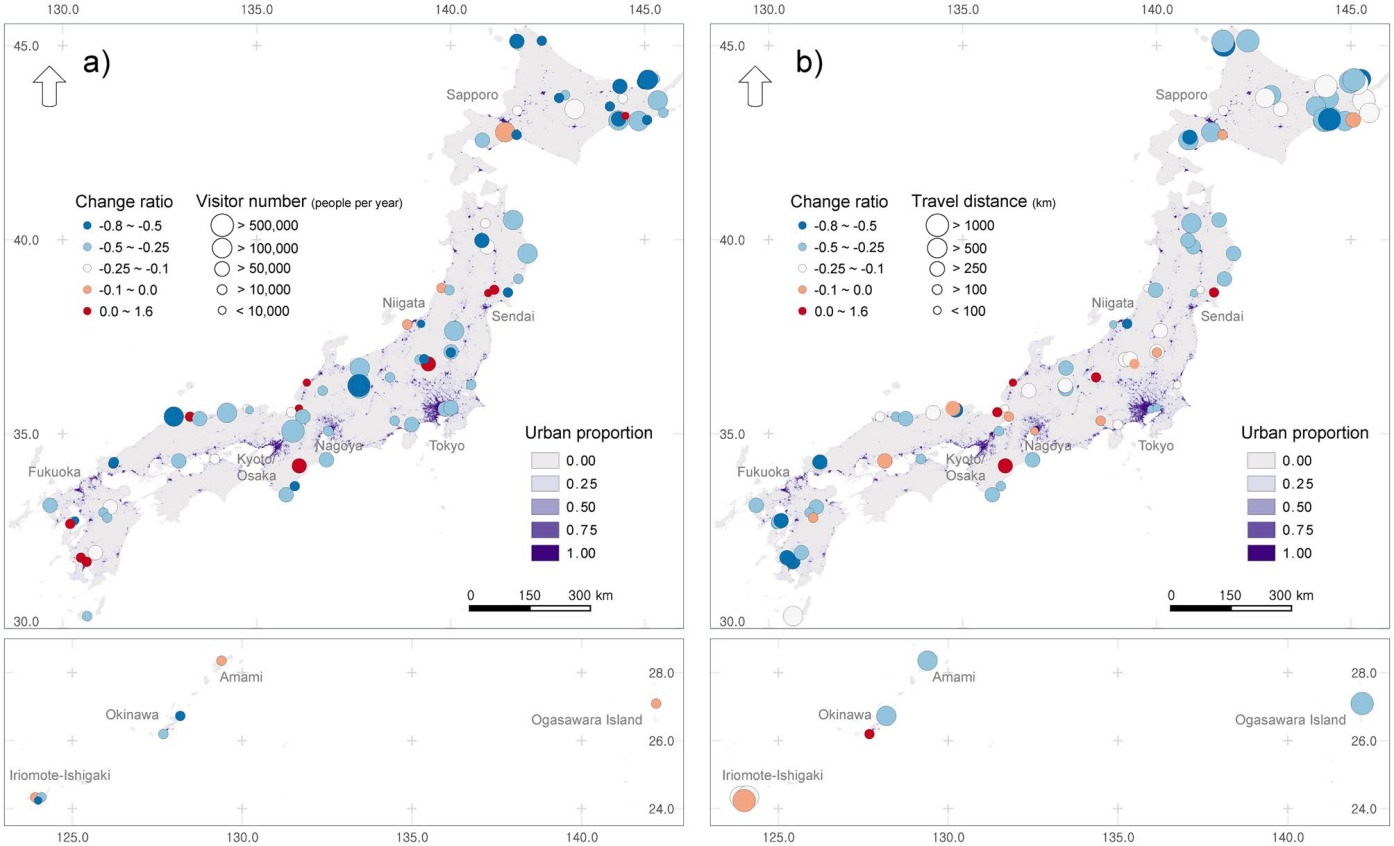
Changes in spatial patterns of visitor characteristics after COVID-19. (**a**) Change ratio of number of visitors and annual number of visitors before COVID-19, (**b**) Change ratio of travel distance and mean travel distance before COVID-19. The background color of the map indicates the urban share on the 1 km grid.

Spatial mapping of the relative change in visitation patterns further explained the visitation responses estimated from the GAM response curve (Fig. 4a,b). Although the site-level response was more complicated, depending on specific site conditions and regional characteristics, conservation sites close to the peri-urban boundary tended to have higher visitation or maintain visitation levels similar to the preCOVID period (Fig. 4a). More remote protected areas, which are more distant from major cities, showed a large decrease in travel distance during COVID-19 (Fig. 4b).

## Discussion

The continuous streaming nature of mobile phone data provides more opportunities to assess the unexpected impacts of natural or social events with high uncertainty that could affect the overall quality of ecosystem services^10^. Our result robustly highlighted the temporal dynamics of visitor patterns in protected areas as a social indicator of realized recreational services after the COVID-19 outbreak. We observed an abrupt decrease after the initial COVID period and a rapid recovery of visitor numbers during the prolonged period of the COVID-19 pandemic (i.e., after the 2nd wave). Increased visitation to protected areas after the COVID-19 outbreak has been consistently reported in several countries with different levels of social countermeasures^14,27,28^.

Comparatively, this characteristic recovery pattern was also reflected in the temporal trend of public interest in protected areas. For example, a global culturomics study using the relative volume of Google searches for national parks similarly captured a sharp decline and recovery in public interest in the early COVID-19 period^29^. The recovery of visitor numbers, even with the increasing peaks of COVID-19 cases, may indicate a higher public demand for the natural environment and the important role of recreational ecosystem services in protected areas with their physical and mental health supports^30,31^, that are often limited in urban areas^32^. These phenomena have been observed during other pandemics. For example, past experience during SARS (Severe Acute Respiratory Syndrome) showed that social stress and frustration levels increased rapidly during quarantine periods^13^, which may have increased public demand for outdoor recreation, although there is little evidence to support these phenomena. Open spaces in protected areas can satisfy public demand for interaction with the natural environment without severely violating protective measures such as social distance restrictions^13,33^. This suggests that such open spaces in protected areas can enhance community resilience while maintaining human well-being through interactions with nature.

The overall size effect of conservation area on annual visitor characteristics was not significant in the GAM results. However, we observed that the temporal recovery of visitor numbers and travel distance after the 1st peak wave of COVID-19 was slightly faster in smaller protected areas (< 150 km^2^) than in medium or large protected areas (Fig. 2c,f). Google community mobility reports showed the similar patterns of change in visitors, but the different magnitudes during the 1st wave periods (https://www.google.com/covid19/mobility). From Google's mobility reports, the magnitude of the decline in park visits was much smaller compared to our results, which focused on national parks and Ramsar sites. Because Google's mobility report includes broader types of regional parks (i.e., small urban parks), both findings highlight the high resilience and short recovery time of visitors to small green spaces (e.g., parks, botanical gardens, and nature reserves) during the pandemic. Moreover, even in large protected areas, the average travel distance remained lower than in the pre-COVID year, as the relative proportion of long-distance travel decreased (Fig. 2d), even after the temporal recovery of visitor numbers under less stringent social measures by the government (e.g., lift of the State of Emergency at prefectural level).

Our results add to the empirical evidence describing the shift in the realized value of protected areas (natural environments) within residential areas during the COVID-19 pandemic^13,32,34^. Specifically, in our study, people preferred protected areas close to their homes while visiting those in less urbanized regions (Figs. 3, 4). A similar pattern has been reported in several US national parks^33^. This public response pattern seems to be closely related to avoidance behavior against the increased probability of COVID-19 infection risk during long-distance travel to remote sites and frequent human contact in a populated urban area^35,36^. During the early stages of COVID, rapid spread of COVID-19 infection and clustered infections in large cities were reported in the mass media. Afterwards, the public's preference for a less crowded park area became higher than in pre-pandemic period^37^. As a result, protected areas that were mainly used by people living in the proximate distance were more adaptable to the pandemic situation with increased visitation. On the other hand, protected areas that were largely dependent on distant travelers for their operating costs or management became more vulnerable to a prolonged pandemic event. This is in contrast to pre-COVID visitation patterns, where remote conservation areas with unique ecosystem types and convenient transportation attracted more visitors through long-distance transportation networks^38,39^.

Our findings suggest preliminary conceptual clues that may support conservation management planning in uncertain pandemic situations. First, the establishment of sufficient conservation areas would satisfy the increased public demand for less populated natural open spaces and enhance the resilience of human well-being. In particular, we believe that increased conservation capacity will contribute to the flow of cultural ecosystem services (e.g., recreational ecosystem services) to regional society through intimate relationships with nature during the pandemic situation. Second, the scale diversity of conservation areas will help maintain higher resilience and recovery of recreational ecosystem services during the pandemic. The development of large areas for natural ecosystems is essential, but requires a lot of time and effort to implement in the developed urban sector. In our study, small protected areas in the peri-urban region showed a more resilient recovery of visitors after the COVID impact. Moreover, some small habitat patches in fragmented landscapes have a higher conservation value for biodiversity against urban development pressures^40,41^. A coordinated network of these small conservation areas may have a higher potential to achieve multiple conservation objectives to protect biodiversity and ecosystem services in times of high uncertainty^42^. Third, maintaining strong links between protected areas and local communities is essential to overcome the prolonged effects of the pandemic. During the COVID-19 outbreak, long-distance travel was limited, while short-distance visits became important activities, highlighting the importance of accessible protected areas in sustaining people's livelihoods. On the other hand, excessive recreational infrastructure to attract long-distance travelers may further jeopardize sustainable conservation efforts in large protected areas if the pandemic situation persists. Finally, adaptive management planning and resource allocation should be expanded based on quantitative visitor monitoring, such as mobile phone GPS data, which can provide timely site information to respond to rapid changes in and around protected areas.

Finally, the present study provides a new possible implication for rapid assessment of visitor characteristics using mobile phone big data. Timely assessment of recreational ecosystem services can guide practical management of protected areas in the post-pandemic era. Efficient proactive management is often hampered by the lack of reliable visitor estimates in conservation areas^43–45^. The high temporal resolution and broad spatial coverage of mobile phone data will allow researchers and practitioners to examine in detail temporal changes or transitions during unexpected events. Some drawbacks, including (1) relatively higher implementation costs of mobile phone data, (2) sampling bias in social groups, (3) ethical compliance, (4) infringement of the personal information, and (5) methodological transparency of raw data management, and (6) uncertain processing bias related to business policies of private companies may be a potential barrier to complement traditional visitor counting methods on a large scale^10,46^. In the case of mobile phone GPS data produced by private mobile operators or internet service providers tends to be more expensive and have less transparent information for data aggregation procedures. However, given the advantages of rapid data collection and the possibility of retrospective analysis after unexpected events, the use of mobile phone data has more advantages for supporting resilient management systems. The rapid development of mobile phone and public data sharing technologies will further facilitate the widespread use of mobile phone data. This site-specific information can help managers efficiently allocate and utilize their management resources according to the dynamic status of visitor demand.

## Methods

### Study sites

We identified and integrated the location of visitor centers in 34 national parks and 52 Ramsar sites in Japan. In order to provide up-to-date site information and educational programs, visitor centers are generally located at the entrance or near central trails, that receive the majority of visitor volume to the protected areas. For spatial filtering of mobile phone GPS data, we digitized 100-200 m buffered boundaries around these visitor centers. After excluding newly opened visitor centers or those under construction from 2019 to 2020 (16 sites), a total of 98 visitor centers were included in the following analysis. We assumed that visitor characteristics near visitor centers could be used as a site-level indicator of overall visitor patterns in protected areas.

### Mobile phone location data

The mobile phone GPS data of this system was collected by au Corporation which has the second largest mobile phone market share in Japan (https://k-locationanalyzer.com/en/). Data collection was done based on an individual consent and commercial contract with au Corporation to collect and share their location data. All personal attribute information was anonymized by KDDI before the aggregation process, and individual user information could not be identified or used by the researchers. Therefore, the use of GPS data in this paper is regarded as a secondary use of the anonymized GPS location data. Ethical approval was exempted for the secondary use of anonymized open data and the study design without direct involvement of research subjects. We further checked that the overall methods used in this study were conducted in accordance with the Helsinki Declaration and related guidelines.

Monthly aggregated visitor information at each location was downloaded from the KDDI Location Analyzer (Fig. 1). In the KDDI Location Analyzer (i.e., data retrieval system), users can search and download the population records within a study boundary (e.g., geofence or polygon). The data frame includes (1) study boundary name, (2) date, (3) time, (4) visitor number, (5) travel distance from their residential area (i.e., town, city level). The original location data of mobile phone users were estimated from their GPS coordinates to an accuracy of < 10 m.

The first officially positive case of COVID-19 was confirmed in Japan on January 15, 2020. From this outbreak date, we defined 2019 as the "before COVID-19" year and 2020 as the "during COVID-19" year. Using the polygon boundary of each visitor center, we queried the monthly number of visitors and visitor attribute information (e.g., travel distance) from January 2019 to December 2020 (24 months). Assuming that a visitor's travel cost increases proportionally with the total travel distance, we used the travel distance (m) from the visitor's residential area to each visitor center as a simplified economic indicator of each visitor's travel cost. To focus on the relative change in visitation pattern during the pandemic periods, we rescaled the monthly visitor counts and mean travel distance using the maximum and minimum values of each visitor center, as described below (Eq. 1).

1 $$ {\text{normalized visitor value }}\left( {{\text{y}}_{{\text{i}}} } \right) \, = \, \left( {{\text{x}}_{{\text{i}}} {-}{\text{ x}}_{{{\text{minimum}}}} } \right) \, / \, \left( {{\text{x}}_{{{\text{maximum}}}} {-}{\text{ x}}_{{{\text{minimum}}}} } \right) $$

X _minimum_ = monthly minimum value of visitor characteristics in 24 months; X _maximum_ = monthly maximum value of visitor characteristics in 24 months; X_i_ = i^th^ value of visitor characteristics (i.e., visitor counts, travel distance)

### Socio-environmental conditions

To identify the influence of surrounding environmental conditions on visitor patterns, six socio-environmental attributes were compared with changes in visitor patterns during the COVID-19 pandemic. We calculated (1) distance from the metropolitan city centers (km), (2) population density (thousand people per 1km^2^), (3) relative proportion of urban land cover (%) in a 10 km buffer area around each visitor center. The distance to the city was calculated by analyzing the distance network between the central points of 24 Japanese metropolitan areas and 98 visitor centers, and then using the shortest distance to the nearest city center (QGIS version 3.20.3). (4) Designated conservation areas (km^2^) were obtained from the National Park Service (https://www.japan.travel/national-parks) and the Ramsar site Information Sheet (https://rsis.ramsar.org). (5) Visitor numbers (people) and (6) mean travel distance of visitor centers (km) before COVID-19 (2019) were also included to understand the effect of visitor capacity and attraction level on visitor changes during COVID-19.

### Statistical analysis

The relationship between the number of visitors, travel distance, and socio-environmental conditions prior to COVID-19 was analyzed by calculating the Spearman correlation coefficient (r_s_) between candidate variables. The difference in visitor characteristics among size groups of the conservation area (i.e., small: < 150 km^2^, medium: < 550 km^2^, large > 550 km^2^) was tested based on the Kruskal–Wallis test. Size group criteria of the conservation area were calculated using the natural breaks function (QGIS version 3.20.3). To account for the nonlinear response of visitor characteristics, changes of visitor characteristics after COVID-19 were estimated using the Generalized Additive Model (GAM). In the GAM, the relative change in visitor numbers and mean travel distance was predicted with prescribed socio-environmental variables, except for population density due to its high correlation with other variables. Size groups of conservation area were included in the GAM as categorical factor variables. Smoothing terms and parametric effects of the fitted GAM were estimated and visualized using the *mgcv* and *mgcViz* packages in R (version 4.0.5)^47,48^. The performance of the GAM was compared using adjusted R-squared value.

### Supplementary Information

Supplementary Information 1.

## Data Availability

The mobile phone GPS data supporting the results of this study are available from the KDDI Corporation (Japan) but restrictions apply to the availability of these data, which were used under license for the current study, and so are not publicly available. Data are however available from the authors upon reasonable request subject to a permission from the KDDI Corporation. Location data (vector) of visitor centers are available from the open data repository (https://doi.org/10.5281/zenodo.10066858). The boundaries of protected areas are available from the National Park Service website (https://www.japan.travel/national-parks) and the Ramsar site Information Sheet (https://rsis.ramsar.org). All socio-environmental variables used in this study are available from the data distribution portal of the Ministry of Land, Infrastructure, Transport and Tourism of Japan (https://nlftp.mlit.go.jp/ksj/).

## Code availability

The code used in this study is available on request from the corresponding author. The code is written in R and is available as a R Script.
