# Supplementary material for: Mobile phone data reveals spatiotemporal recreational patterns in conservation areas during the COVID pandemic
Source: Sci Rep. 2023 Nov 20;13:20282. doi: 10.1038/s41598-023-47326-y (PMC10660657; doi:10.1038/s41598-023-47326-y)
Supplement: Supplementary file 1 — Supplementary Information 1. [file 41598_2023_47326_MOESM1_ESM.pdf]

Supplementary & Table & Figure

Supplementary Table 1. Change in visitor characteristics from April to September (1st peak wave) before COVID year (2019) and after COVID year (2020). small: <150 km^2, medium: <550 km^2, Large >550km^2

Visitor characteristics	Size group	Before COVID	During COVID	Kruskal-Wallis H	p
Number of visitors (people)	Large	61782.5 ±18036.6	27035 ±6082.9	8.520	0.004
	Medium	42755.3 ±11121.6	19181.5 ±4439.3	3.454	0.063
	Small	27481.4 ±9150.6	14321.4 ±5245.1	2.621	0.105
Travel distance (km)	Large	210.9 ±23.8	117.2 ±10.7	8.866	0.003
	Medium	525.4 ±77.94	276.3 ±51.2	8.962	0.003
	Small	159.9 ±32.4	95.7 ±19.4	1.596	0.207

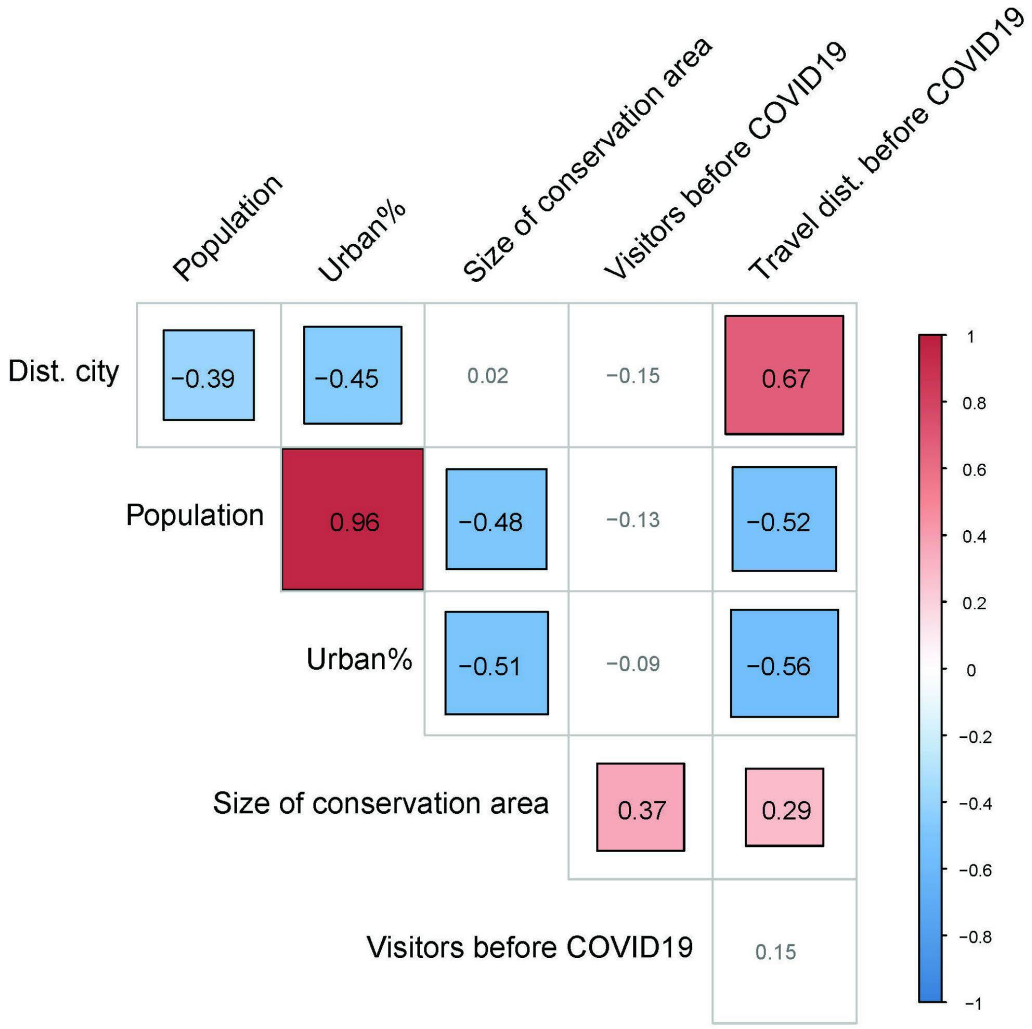

Supplementary Figure 1. Relationship between number of visitors, travel distance, and socio-environmental conditions around visitor centers prior to COVID-19. The value in the matrix is the Spearman correlation coefficient (rs) between the variables. The color scale and square size are proportional to the Spearman coefficient. Only significant coefficients are shown with color scale. Travel dist.: Travel distance.

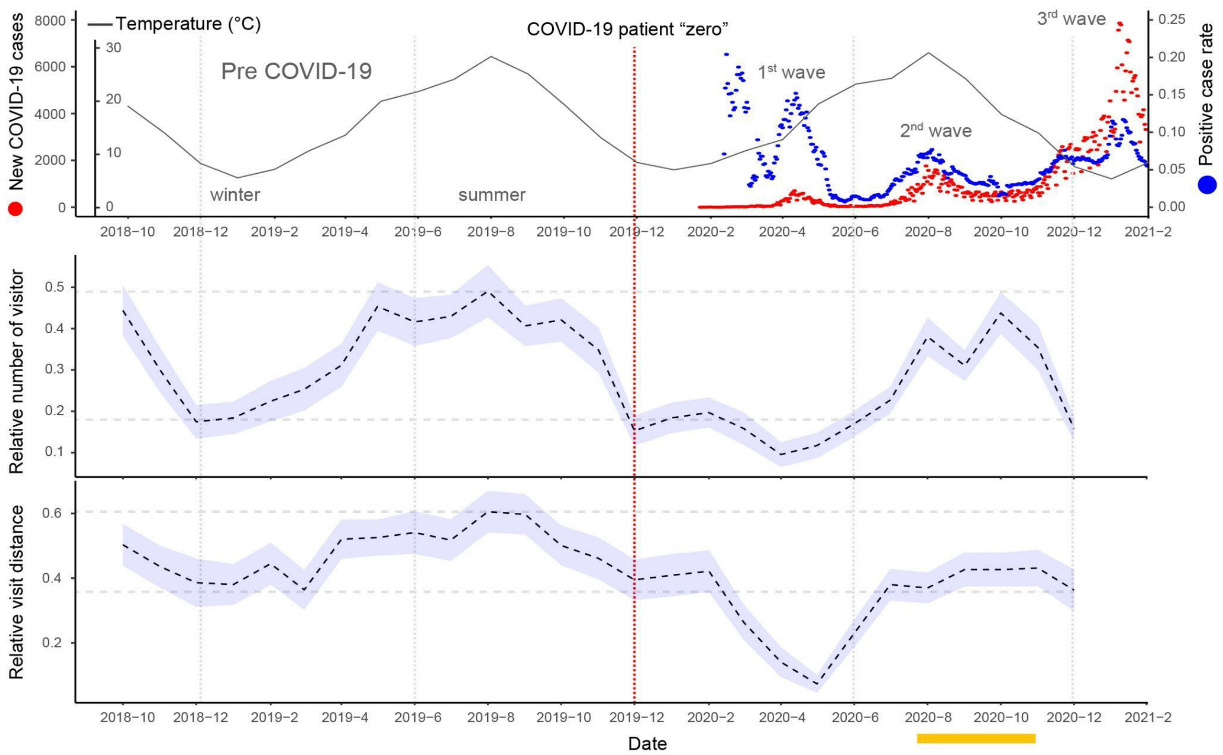

Supplementary Figure 2. Temporal comparison of new COVID-19 cases and visitor characteristics at study sites. Colors indicate 95% confidence interval. The first official COVID-19 case in Japan was reported in December 2019.

Other supplementary information (provided by the other forms)

- Point vector format: Point location of visitor centers in National Parks and Ramsar sites in Japan (available at https://doi.org/10.5281/zenodo.10066858)
